# A novel cold-adapted esterase from *Enterobacter cloacae*: Characterization and improvement of its activity and thermostability via the site of Tyr193Cys

**DOI:** 10.1186/s12934-018-0885-z

**Published:** 2018-03-19

**Authors:** Haofeng Gao, Chanjuan Li, Ramesh Bandikari, Ziduo Liu, Nan Hu, Qiang Yong

**Affiliations:** 1 College of Light Industry Science and Engineering, Nanjing Forestry University, 159 Longpan Road, Nanjing, 210018 China; 2 College of Biotechnology and Pharmaceutical Engineering, Nanjing Tech University, 30 Puzhu South Road, Nanjing, 211800 China; 3 College of Life Science and Technology, State Key Laboratory of Agricultural Microbiology, Huazhong Agricultural University, Wuhan, 430070 China

**Keywords:** Random mutagenesis, Site-direct mutagenesis, Enzymatic activity, Thermo**-**stability

## Abstract

**Background:**

In industries lipolytic reactions occur in insensitive conditions such as high temperature thus novel stout esterases with unique properties are attracts to the industrial application. Protein engineering is the tool to obtain desirable characters of enzymes. A novel esterase gene was isolated from South China Sea and subjected to a random mutagenesis and site directed mutagenesis for higher activity and thermo**-**stability compared to wild type.

**Results:**

A novel esterase showed the highest hydrolytic activity against *p*-nitrophenyl acetate (*p*NPA, C2) and the optimal activity at 40 °C and pH 8.5. It was a cold-adapted enzyme and retained approximately 40% of its maximum activity at 0 °C. A mutant, with higher activity and thermo**-**stability was obtained by random mutagenesis. Kinetic analysis indicated that the mutant Val29Ala/Tyr193Cys shown 43.5% decrease in *K*_*m*_, 2.6-fold increase in *K*_*cat*_, and 4.7-fold increase in *K*_*cat*_/*K*_*m*_ relative to the wild type. Single mutants V29A and Y193C were constructed and their kinetic parameters were measured. The results showed that the values of *K*_*m*_, *K*_*cat*_, and *K*_*cat*_/*K*_*m*_ of V29A were similar to those of the wild type while Y193C showed 52.7% decrease in *K*_*m*_, 2.7-fold increase in *K*_*cat*_, and 5.6**-**fold increase in *K*_*cat*_/*K*_*m*_ compared with the wild type. The 3-D structure and docking analysis revealed that the replacement of Tyr by Cys could enlarge the binding pocket. Moreover Y193C also showed a better thermo-stability for the reason its higher hydrophobicity and retained 67% relative activity after incubation for 3 h at 50 °C.

**Conclusions:**

The superior quality of modified esterase suggested it has great potential application in extreme conditions and the mutational work recommended that important information for the study of esterase structure and function.

**Electronic supplementary material:**

The online version of this article (10.1186/s12934-018-0885-z) contains supplementary material, which is available to authorized users.

## Background

Esterases are ubiquitous enzymes widely distributed in plants, animals and microorganisms, and they represent a diverse group of hydrolases catalyzing the cleavage and formation of ester bonds [1]. In contrast to lipases, esterases hydrolyze soluble fatty acid esters without any interfacial activation [2] and display a typical Michaelis–Menten behavior [3].

In recent years, esterases have been widely used in food production, detergent products, pharmaceuticals, perfumes, degradation of pollutants and the synthesis of optically pure compounds [4, 5] owing to their broad array of substrate specificity and versatility in the reactions they catalyze [3]. Esterases with novel properties have a more and broad application prospect: cold-adapted esterases are applied to the industrial reaction at low temperature and benefit energy conservation [6–8]; salt-tolerant esterases are suitable for the reactions under a high salt concentration [9–11]; organic solvent-tolerant esterases/lipases are necessary for the substrates which are insoluble to water and the trans-esterification reaction in biodiesel production [5, 12, 13]. Nevertheless, there have been only a few reports available about these esterases with novel properties, implying the necessity to find more novel esterases.

Enhancing enzymatic activity of an esterase was also an important for versatility. According to previous reports, many mutations are discovered by screening functions such as activity and thermal stability [14–17]. All information of mutations can facilitate the discovery of an esterase with more application value.

In this study, a gene of a novel esterase, *Lip*, was cloned from the marine bacterium *Enterobacter cloacae* and expressed in *Escherichia coli* (*E. coli*). The esterase showed high hydrolytic activity at low temperature and tolerance to organic solvents. By random mutagenesis, we obtained a double mutant with enhanced activity, V29A/Y193C, and single mutants V29A and Y193C were individually obtained by site-directed mutagenesis. Kinetic analysis of V29A/Y193C, V29A and Y193C suggested that decrease of *K*_*m*_ and increase of *K*_*cat*_ are only attributed to Y193C. Besides, Y193C showed better thermal stability than the wild type.

## Results

### Cloning and sequence analysis

**Fig. 1:**
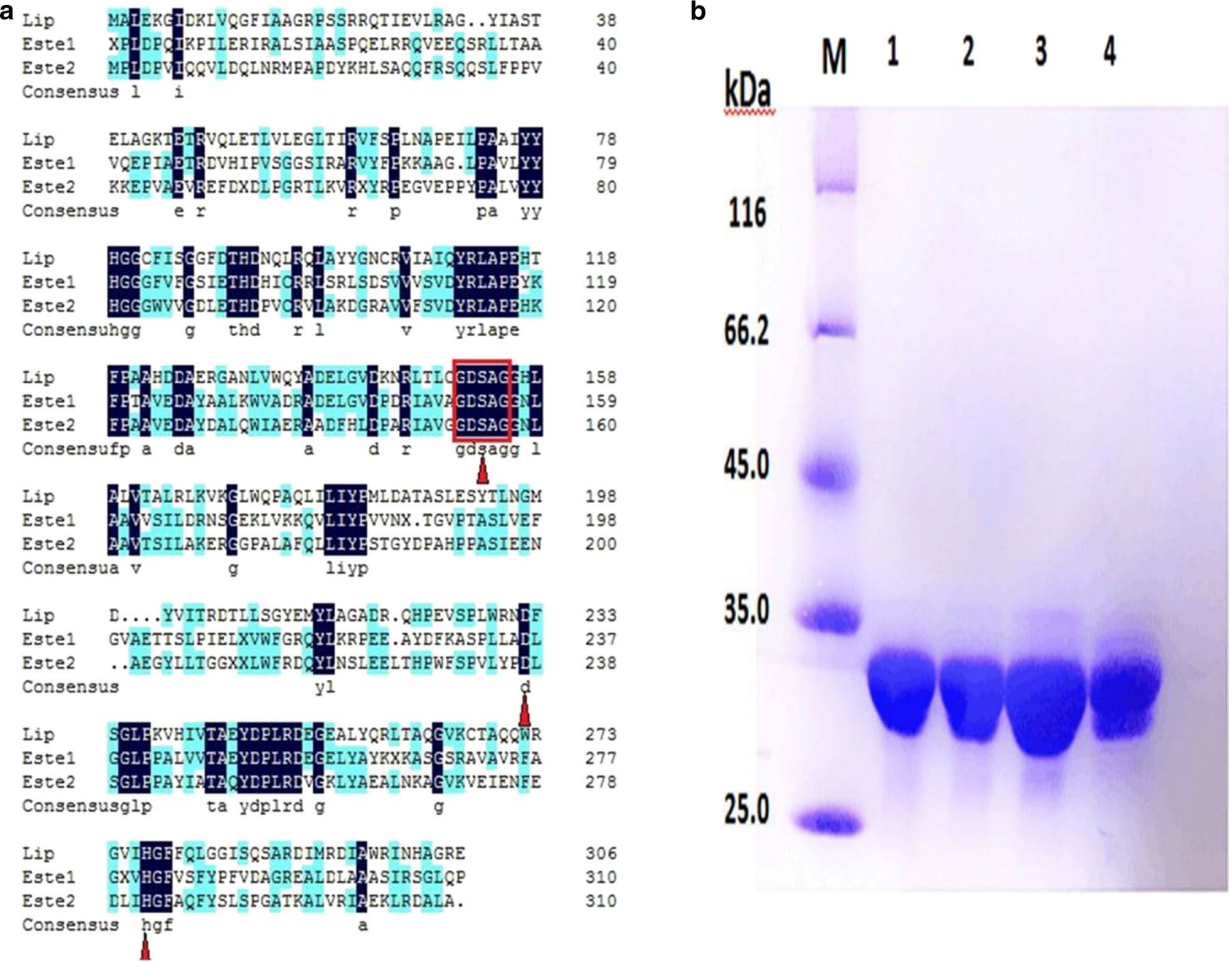
**a** Multiple sequence alignments of *Lip* and the other two esterases (Este1 PDB: 2C7B_A, Este2 PDB: 1EVQ_A). The strictly conserved residues are filled with blue and the catalytic triads are indicated by the red triangles and the conserved motif is indicated by the red box. **b** SDS-PAGE analysis of *Lip* and mutants (10 µl; protein concentration 7 µg). M: Protein molecular weight marker; 1: The wild type *Lip* protein; 2: The mutant V29A/ Y193C protein; 3: The mutant V29A protein; 4: The mutant Y193C protein

The esterase gene, *Lip*, was successfully cloned from *E. cloacae* with a length of 921 bp encoding 306 amino acid residues with a calculated molecular mass of 33.9 kDa. No signal peptide in this sequence was predicted by the SignalP4.1 Server. The catalytic triads and conserved motifs of Lip were displayed by the multiple sequence alignment with a new thermophilic and thermostable carboxylesterase Este1 [PDB: 2C7B_A] from a metagenomics library (identity: 31%) and a thermophilic carboxylesterase Este2 [PDB: 1EVQ_A] from *Alicyclobacillus acidocaldarius* (identity: 32%). The catalytic triads consisted of Ser 153, Asp 232 and His 277, and the conserved motif was Gly151-X-Ser153-X-Gly155 (Fig. 1a).

### Screening of random mutant library and reverse mutation

The mutant library was screened by high-throughput screening and we acquire the 8000 clones, consequently ensure the activity of each one and the average mutational rate was shown more than 50%. Among them a mutant V29A/Y193C displayed higher catalytic efficiency than the wild type. To investigate the effect of each site, two single site mutants V29A and Y193C were constructed and analyzed.

### Expression and purification of *Lip* and mutants

The protein *Lip* and mutants were expressed successfully and purified by the removal of GST Tag. The size (~ 33.9 kDa) was detected by SDS-PAGE and consistent with the value predicted form the deduced amino acid sequence (Fig. 1b).

### Substrate specificity

**Fig. 2:**
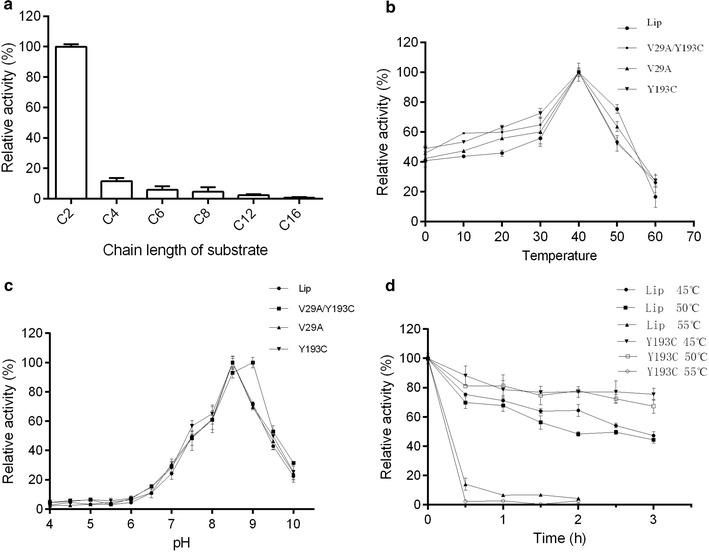
**a** Substrate specificity of purified *Lip*. The activity towards C2 as 100%. **b** Effect of temperature on the activity of *Lip* and mutants. The activity at 40 °C as 100%. **c** Effect of pH on the activity of *Lip* and mutants. Activity of wild type, V29A, Y193C and V29A/Y193C at pH 8.5 and 9 as 100%. **d** Thermo-stability of *Lip* and Y193C. The residual activity was measured at 40 °C by collecting enzymes every 30 min. The specific activity without incubation was defined as 100%

*Lip* showed the maximum hydrolytic activity against *p*-NP acetate (C2), slight activity toward C4, C6, C8 and C12, and no activity toward *p*-NP palmitate (C16). The results indicated that *Lip* is esterase rather than lipase (Fig. 2a). The mutant Y193C was also similar results as the wild type (Additional file 1: Figure S1).

### Biochemical characterization of *Lip* and mutants

The activity of *Lip* and mutants was the highest at 40 °C, decreased radically at the temperature above 50 °C, and maintained 40–50% relative activity at 0 °C (Fig. 2b). The data indicated that *Lip* was a cold-adapted esterase. V29A/Y193C showed the highest activity at pH 9.0 while *Lip*, V29A, Y193C showed the optimal activity at pH 8.5 (Fig. 2c). Y193C performed better in thermal stability than *Lip* when the temperature was less than 50 °C, retaining 67% relative activity after incubation for 3 h at 50 °C compared to 44% for the wild type. Both *Lip* and Y193C retained more than 50% of the maximum activity after incubation at the temperature below 50 °C for 3 h and lost activity in 30 min at 55 °C (Fig. 2d).

**Fig. 3:**
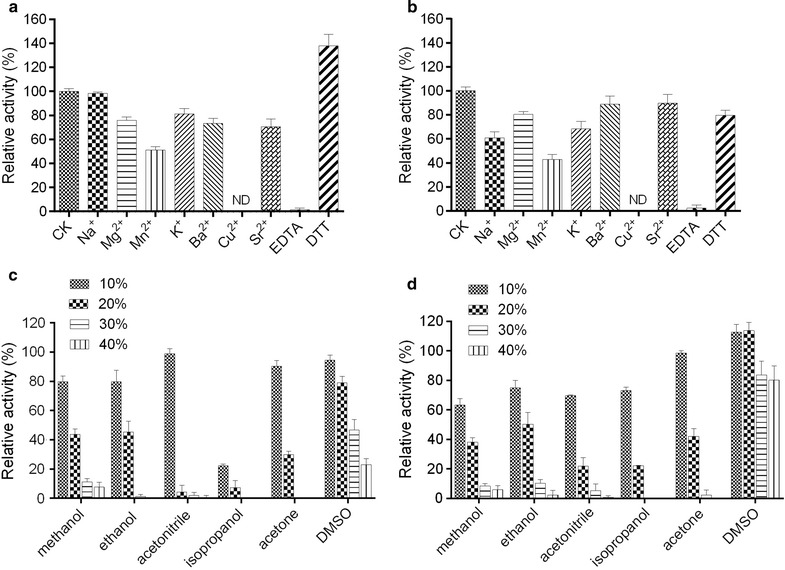
**a** Effects of metal ions and reagents on activity of the wild type *Lip*; **b** effect on activity of the mutant Y193C; **c** effect of organic solvents and detergents on activity of the wild type *Lip*; **d** effects of organic solvents and detergents on activity of the Y193C. The activity without additional metal ions and reagents was defined as 100%. ND means not detected

The effect of metal ions and reagents on activity was shown in Fig. 3a and b. Five mMNa^+^ inhibited the activity of Y193C (a decrease of 40%), but showed no effect on *Lip*. The activity of *Lip* was slightly inhibited by Mg^2+^, K^+^, Ba^2+^ and Sr^2+^, which was similar to that of Y193C. The activities of both *Lip* and Y193C were strongly inhibited by Mn^2+^ (retaining less than 50%) and EDTA (retaining less than 5%), and were completely undetectable by Cu^2+^. DTT could stimulate the activity of *Lip* (an increase of 38%), but inhibit the activity of Y193C (a decrease of 20%).

*Lip* presented better stability than Y193C in 10% of methanol, ethanol and acetonitrile, but Y193C showed better stability in 10% isopropanol (73% relative activity) and higher concentration of DMSO (80% relative activity). Both *Lip* and Y193C showed no activity in butyl alcohol, Tween-20, Tween-80, and SDS (Fig. 3c, d).

### Kinetic parameters

Kinetic parameters of *Lip* and mutants were determined under optimal conditions (Table 1). The mutant V29A/Y193C showed a 43.5% decrease in *K*_*m*_, a 2.6-fold increase in *K*_*cat*_, and a 4.7-fold increase in *K*_*cat*_/*K*_*m*_. V29A showed a similar value of *K*_*m*_, *K*_*cat*_, and *K*_*cat*_/*K*_*m*_ to that of the wild type. Y193C showed 52.7% decrease in *K*_*m*_, a 2.7-fold increase in *K*_*cat*_, and a 5.6-fold increase in *K*_*cat*_/*K*_*m*_. The results indicated that the site Y193C, rather than V29A, played a significant role in the increased catalytic efficiency of mutant V29A/Y193C.

**Table 1:** The kinetic parameters of the wild type and mutants

Enzyme	Protein (μg/ml)	*K*_*m*_ (mM)	*K*_*cat*_ (s^−1^)	*K*_*cat*_/*K*_*m*_ (s^−1^ mM^−1^)
Lip	203.41 ± 3.26	0.643 ± 0.03	9.8 ± 0.09	15.24 ± 0.21
V29A/Y193C	197.29 ± 2.54	0.363 ± 0.03	25.8 ± 0.16	71.08 ± 1.03
V29A	173.23 ± 2.17	0.624 ± 0.05	10.2 ± 0.11	16.35 ± 0.31
Y193C	183.42 ± 2.91	0.304 ± 0.04	26.1 ± 0.15	85.87 ± 1.62

Data are given as mean values ± S.D. All the assays were performed at the optimal pH and temperature for the protein being studied

### Homology modeling

**Fig. 4:**
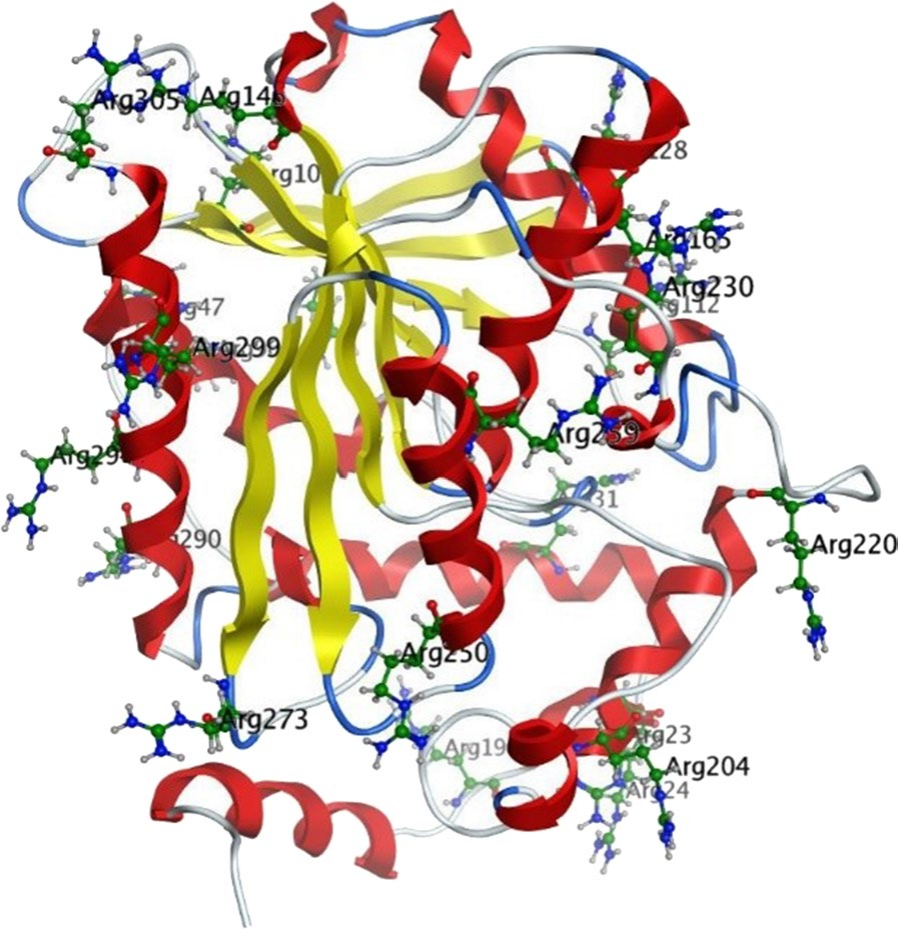
Overall 3-D structure of the wild type *Lip*. For clarity, only arginine side chains (charged residues) were shown

**Fig. 5:**
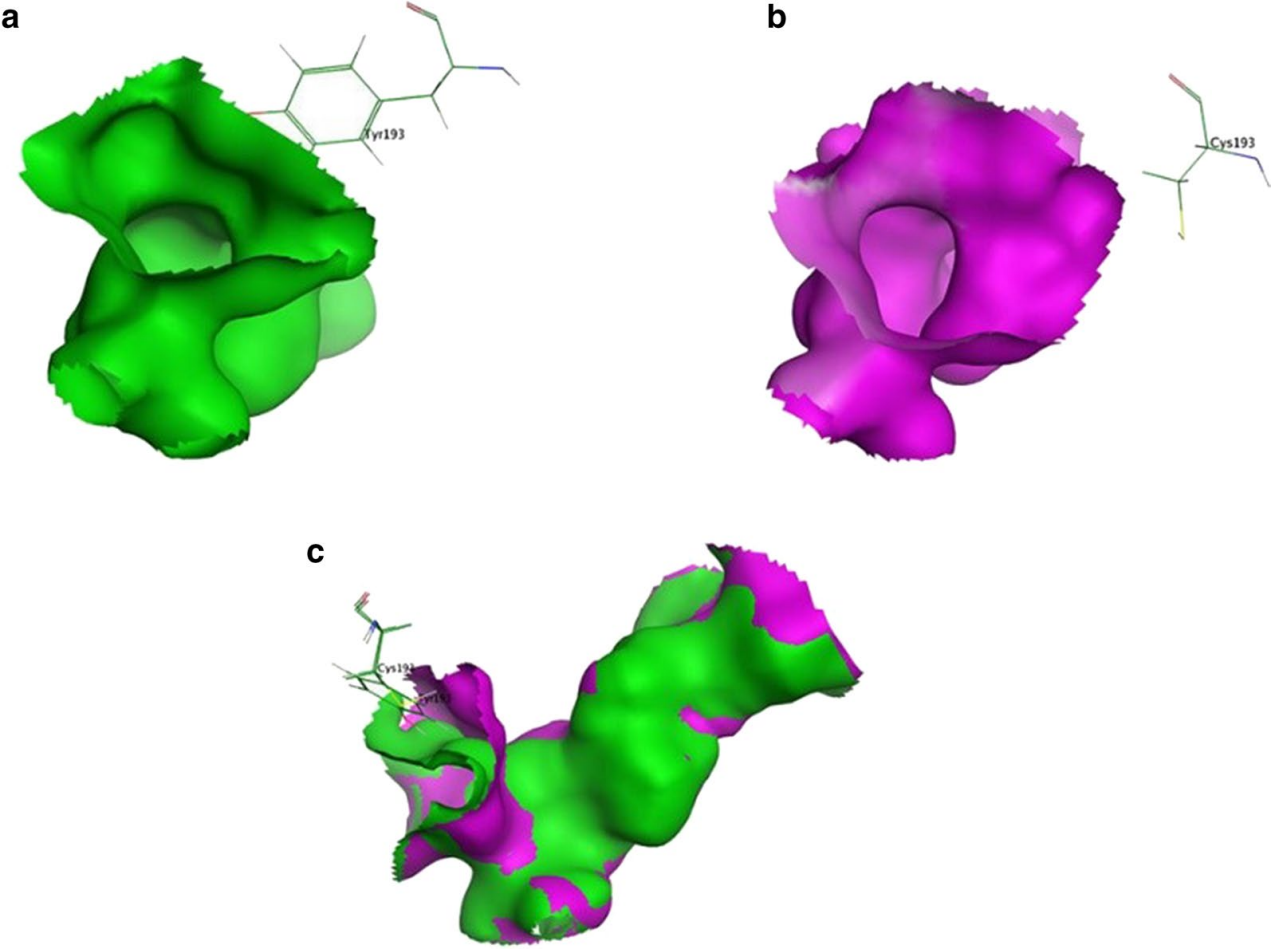
Binding pockets of WT and mutant. **a** The binding pocket of the wild type *Lip*; **b** the binding pocket of the mutant Y193C; **c** the superposition of binding pockets of *Lip* (green color) and Y193C (purple color)

**Fig. 6:**
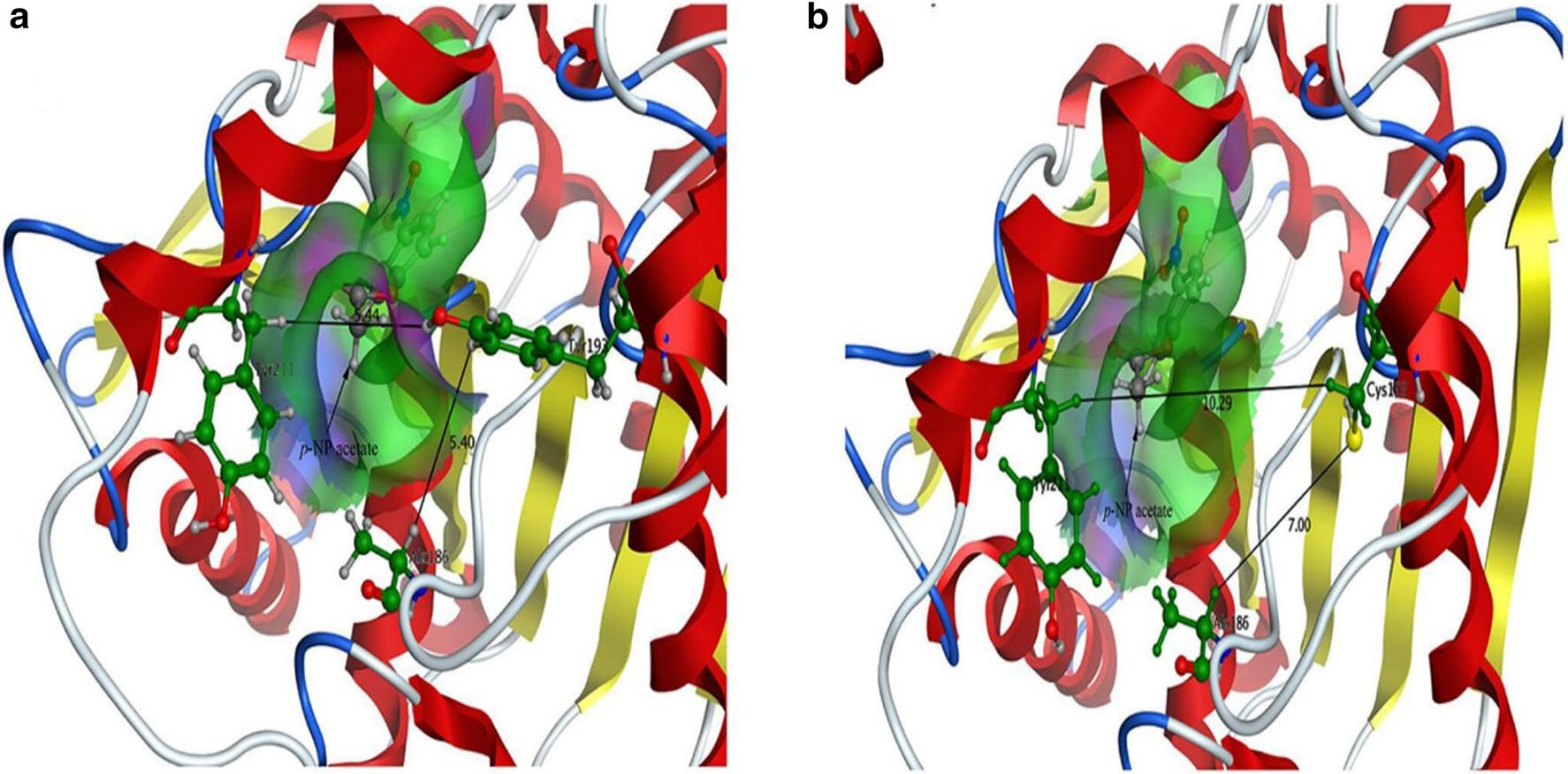
The distances between residue 193 and residue 211 and between residue 193 and residue 186. **a** Docking analysis of *Lip* and *p*-NP acetate complex showing the position of *p*-NP acetate in active cavity and location of residue Tyr_193_. **b** Docking analysis of Y193C and *p*-NP acetate complex showing the position of *p*-NP acetate in active cavity and location of residue Cys_193_

The 3-D models of *Lip* and mutants were constructed based on an alpha/beta hydrolase enzyme [PDB: 5JD4_A] from the metagenome of Lake Arreo (33.77% identity), and the structural features of *Lip* are shown in more detail (Fig. 4). To explore the effects of Y193C on catalytic activity, docking analysis was performed based on the homology model. It can be seen that Y193C had a larger binding pocket (Fig. 5) because of the replacement of tyrosine by cysteine, and the distance between residue 193 and residue 211 increased from 5.44 to 10.29 Å while the distance between residue 193 and residue 186 increased from 5.4 to 7 Å (Fig. 6).

## Discussion

In this study, a novel esterase gene was successfully cloned from *E. cloacae*, expressed in *E. coli* BL21 and the protein was purified. *Lip* showed the catalytic activity of short chain substrate and the highest catalytic activity of *p*-nitrophenyl acetate (C2), revealing that *Lip* was an esterase instead of a lipase.

The characterization of *Lip* revealed that it was a cold-adapted esterase. *Lip* could retain approximately 50% relative activity even at 0 °C and inactivate quickly at 55 °C (Fig. 2). These characteristics were similar to those of other reported cold-adapted esterases. For instance, a cold-adapted EstK cloned from *Pseudomonas mandelii* [18–20]. Phthalate ester hydrolase gene was identified from biofilms of a wastewater treatment plant shows activity at low temperatures [21]. Est10 from *P. pacificensis* showed the highest activity at 25 °C and retained 55% relative activity at 0 °C [22]. The optimal temperature of rEst97 was 35 °C and retained 12% relative activity at 0.5 °C [23]. MHlip displayed optimal activity at 33 °C, and maintained 20% relative activity at 4 °C [24].

The increase of conformational flexibility was usually considered as a significant factor of cold-adaptation, which appears to be obtained by a high ratio of Gly residues: less Pro and Arg and more Ser and Met [25–28]. Similarly, the decrease of the Pro content and the ratio Arg/Arg + Lys could make lipases active at low temperature [29]. *Lip* has obviously a higher percentage of small amino acids such as Ala (10.5%) and Gly (9.2%) than Pro (3.9%) and Arg (7.2%), which was consistent with previous reports. However, the ratio Arg/Arg + Lys of *Lip* (0.73) was higher than rEST97 (0.56) and MHlip (0.53), but *Lip* had better cold-adaptation, which seems to contradict previous report. Additionally, the majority of Arg residues were distributed on the surface of the protein (Fig. 4), and the abundance of charged residues on the surface could enhance conformational flexibility and ability of interaction with the solvent [30].

According to previous reports, mutation plays an important role in increasing catalytic properties [31–34]. In this study, a double mutant with enhanced enzymatic activity was obtained by random mutagenesis with error-prone PCR. To investigate the effect of each site, two single site mutants V29A and Y193C were constructed and analyzed. The result showed that Y193C was a positive mutant. The 3-D structure and docking analysis revealed that the enlargement of the binding pocket was caused by the replacement of Tyr by Cys (Fig. 5). The replacement of Y193C might remodel the arrangement of residues and change the backbone and side chain, lead to the alternation of the secondary structure and the shape of the binding pocket, and finally change the catalytic activity of enzyme [35]. Besides, Tyr193 is closely located near the binding pocket, and the long amino acid side chain seems to reduce substrate access from the binding to the catalytic cavity, the side chain of Cys was much shorter than Tyr (Fig. 5). For all the reasons above, the distances between residue 193 and residue 211 and between residue 193 and residue 186 increased 4.85 and 1.6 Å respectively (Fig. 6). Accordingly, benefiting from the decrease of the steric hindrance, the substrate could enter the central binding pocket more easily and the product could release conveniently, leading to the enhanced catalytic activity of Lip.

**Fig. 7:**
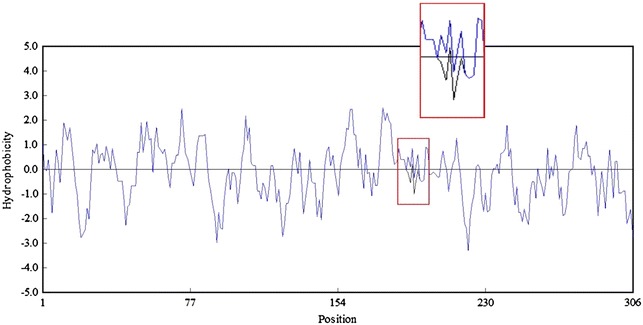
Hydrophobicity of the wild type *Lip* (black line) and Y193C (blue line). The differences were shown in the red box

The better performance of Y193C than the wild type in thermo-stability is probably attributed to the replacement of Tyr by Cys and the replacement may increase the probability of forming a disulfide bond. According to previous reports, Cys could be classified as thermo-labile due to its tendency to undergo deamidation or oxidation at high temperature [36, 37] and protein thermo-stability could be improved by introducing disulfide bond. In addition, thermophilic protein was substantially more hydrophobic [38] because hydrophobic effect was the dominant driving force in protein folding [39]. Therefore, the replacement of Tyr by Cys increased hydrophobicity of Y193C relative to the wild type (Fig. 7), resulting in better thermal stability.

Five mM Na^+^ inhibited the activity of Y193C slightly while the enzyme activity was not affected by Mg^2+^, K^+^, Ba^2+^ and Sr^2+^. The activities of *Lip* and Y193C were strongly inhibits by Mn^2+^ whereas Cu^2+^ showed no activity. Similarly Mohamed [40] reported the inhibitory effect of Mn^2+^ on esterases EII and EIII. Al Khudary [41] and Metin [42] results also suggested that EstO and HBB-4 esterase were completely inhibited by addition of Cu^2+^. The strongly impact of EDTA on catalytic activity indicate that *Lip* and Y193C are metal dependent enzymes. The results were consistent with EstO and Hbb-4 esterase, Metin [42] stated that some ions have a structural role rather than being involved in catalytic activity. DTT could stimulate the activity of *Lip*, but inhibit the activity of Y193C. While the replacement of Tyr by Cys may increase the probability of forming a disulfide bond, the disulfide bond may inhibit the activity of the enzyme.

Both *Lip* and Y193C showed activity in low concentration organic solvents, suggesting their potential use in organic synthesis, non-aqueous reactions and synthesis of esters [43, 44]. Meanwhile, Y193C retained 80% relative activity in 40% DMSO, whereas *Lip* maintained only about 29%, implying a wider application of Y193C than the wild type. The result supports the assumption that thermostable proteins tend to have high tolerance to organic solvents [45].

## Conclusions

In this study, a novel cold-adapted esterase *Lip* from *E. cloacae* was successfully cloned and expressed. It showed not only considerable hydrolytic activity at low temperature, but also organic solvent tolerance. Moreover, we obtained a mutant Y193C with enhanced hydrolytic activity and thermostability. These results provide useful information about the relationship between structure and function of esterases.

## Methods

*p*-nitrophenyl acetate (*p*NPA, C2), *p*-nitrophenyl butyrate (*p*NPB, C4), *p*-nitrophenyl hexanoate (*p*NPH, C6), *p*-nitrophenyl caprylate (*p*NPC, C8), *p*-nitrophenyl laurate (*p*NPL, C12), and *p*-nitrophenyl palmitate (*p*NPP, C16) were purchased from Sigma-Aldrich (St Louis, MO, USA). Taq DNA polymerases, DNA markers and restriction enzymes were purchased from TAKARA (Dalian, China). T4 ligase was purchased from New England Bio Labs lnc. (Singapore). The primers were synthesized by TSINGKE Co. (Wuhan, China). Gel purification kit and plasmid extraction kit were purchased from AXYGEN (USA). All the other chemicals and buffers used were of high purity and analytical grade.

### Strains, vectors and medium

The marine *E. cloacae* strain ZS825 (CCTCC AB2017124) was isolated from the surface seawater in the coastal area of Fujian, China and was grown in HLB (High-salt Luria–Bertani medium, NaCl 2%, peptone 1% and yeast extract 0.5%) with shaking at 180 rpm, 28 °C. *E. coli* strains DH5α and BL21 (DE3) used as hosts for gene cloning and protein expression respectively, were grown in LB (Luria–Bertani medium, NaCl 1%, peptone 1% and yeast extract 0.5%) with shaking at 180 rpm, 37 °C. The plasmid pGEX-6P-1 (GE Healthcare, USA) was used as vector for gene cloning and protein expression.

### Gene cloning

The primers of gene *Lip* (Accession No. MF101724) were designed by putative gene from the sequences of *E. cloacae* strain AR_0002 (Accession No. CP018814.1) were shown in (Table 2).

**Table 2:** Primers were used for random and site directed mutation

1	*Lip*-F: CGCGGATCCATGGCACTGGAAAAGGGT (with *Bam*H I restriction site underlined)
2	*Lip*-R: CCGCTCGAGTCACTCTCGCCCGGCA (with *Xho*I restriction site underlined)
3	Lip-V29A-F: GCCAGACAATTGAGG*C*ACTACGAGCA
4	Lip-V29A-R: *G*CCTCAATTGTCTGGCGACGCGATG
5	Lip-Y193C-F: CCAGTCTTGAAAGCT*G*TACCCTCAAT
6	Lip-Y193C-R: *C*AGCTTTCAAGACTGGCCGTAGCGT

The genomic DNA of *E. cloacae* was used as a template. The program of PCR amplification was: 95 °C for 4 min; 34 cycles of 95 °C for 30 s, 58 °C for 30 s, and 72 °C for 55 s; 72 °C for 10 min. The amplified products and pGEX-6P-1 were purified and digested with *Bam*H I/*Xho*I, and then fused to generate the recombinant plasmid pGEX-6P-*Lip*. The recombinant plasmid was transformed into *E. coli* BL21 (DE3) for protein expression and purification.

### Construction of mutant library

The random mutant library of *Lip* gene was constructed by error-prone PCR [46]. The 50 μl PCR mixture contained 20 ng of the recombinant plasmid pGEX-6P-*Lip*, 0.2 mM dNTPs, 0.2 mM MnCl_2_, and 0.4 μM primers containing *Lip*-F and *Lip*-R, and 2.5 units of Taq DNA polymerase. The error-prone PCR reaction was carried out under similar conditions to those for *Lip* gene. The purified PCR products were digested with *Bam*H I/*Xho*I and then ligated into pGEX-6P-1 with the same digestion system. The recombinant plasmid was transferred into *E. coli* DH5α, and then the cells were spread on LB agar plate containing 100 μg ml^−1^ ampicillin and incubated at 37 °C.

### Screening of library

The colonies were grown on LB plates and picked up with sterile toothpicks. Then the colonies were grown in 96-deep well plates containing 600 μl LB medium with 100 μg ml^−1^ ampicillin. After incubation at 37 °C for 20 h, the mixture was supplemented with 200 μl LB medium containing 100 μg ml^−1^ ampicillin, 0.4 mM IPTG and T7 phage, and then incubated for another 6 h at 18 °C. Finally, the supernatant from each well was collected for activity assay.

### Site-directed mutagenesis

Fast Mutagenesis System (TRANSGEN BIOTECH, China) was used for site-directed mutagenesis, and two pairs of primers (Table 2) were designed based on the result of the screening of mutant library.

The wild-type recombinant plasmid pGEX-6P-*Lip* was used as a template. The PCR program was: 94 °C for 5 min; 25 cycles of 94 °C for 20 s, 55 °C for 20 s, and 72 °C for 30 s; 72 °C for 10 min. One μl *Dpn*I was added into the PCR products for digesting the template, and after incubation at 37 °C for 1 h, the mixture was transformed into *E. coli* DH5α.

### Expression and purification of *Lip* and mutants

The *Lip* and mutants were expressed in *E. coli* BL21, and cells were induced by adding 0.1 mM isopropyl-β-d-thiogalactopyranoside (IPTG) when the optical density of the culture reached 0.6 at OD_600_. After incubation for another 16 h at 18 °C, the cells were collected and then washed twice with PBS buffer (0.8% NaCl, 0.02% KCl, 0.027% KH_2_PO_4_, 0.142% Na_2_HPO_4_). Further, the cells were disrupted by French pressure cell treatment and the crude enzyme was obtained by centrifuging at 4 °C. The crude enzyme was purified by Glutathione-Sepharose column (GE Healthcare, USA) and 3C protease (PreScission, Pharmacia) was added to remove GST tag. Finally, the protein was analyzed by SDS-PAGE and determined by the Bradford method [47].

### Enzyme activity assay

The esterase activity was determined spectrophotometrically by measuring the production of *p*-nitrophenol. The reaction mixture (final volume, 200 μl) containing 188 μl of Tris–HCl buffer (50 mM, pH8.5), 2 μl of *p*-NP ester (10 mM) and 10 μl enzyme was incubated at 45 °C for 10 min, and the reaction without any enzyme was considered as control. The amount of released *p*-nitrophenol was determined from the optical density at 405 nm.

### Substrate specificity

The substrate specificity of *Lip* was investigated with *P*-NP esters of different chain lengths. The substrates were *p*-nitrophenyl acetate (*p*NPA, C2), *p*-nitrophenyl butyrate (*p*NPB, C4), *p*-nitrophenyl hexanoate (*p*NPH, C6), *p*-nitrophenyl caprylate (*p*NPC, C8), *p*-nitrophenyl laurate (*p*NPL, C12), and *p*-nitrophenyl palmitate (*p*NPP, C16).

### Biochemical characterization of enzyme

The optimal temperature of *Lip* was determined by incubating the reaction mixture in the temperature range of 0–60 °C.

The thermal stability was determined by measuring the residual activity of the enzyme after incubation at 45, 50 and 55 °C.

The optimal pH was determined from 4.0 to 10.0 (4.0–8.0 pH buffers were prepared with phosphate and citrate, and 8.5–10.0 buffers were prepared with borax and NaOH).

The effect of metal ions Na^+^, Mg^2+^, K^+^, Ba^2+^, Mn^2+^, Cu^2+^ and Sr^2+^; and inhibitory additives (EDTA, DTT) on enzyme activity were determined at the final concentration of 5 mM.

The effect of organic solvents (methanol, ethanol, acetonitrile, isopropanol, butyl alcohol, acetone, DMSO) and detergents (Tween-20, Tween-80, SDS) were examined in different final concentrations (10–40%).

### Kinetic parameters

The *K*_*m*_ and *K*_*cat*_ of *Lip* and mutants were determined by measuring the reaction rate in different substrate concentrations (5–400 μΜ) under optimal conditions. The *V*_*max*_ and *K*_*m*_ were estimated by the Lineweaver–Burk plot method using the Graph pad Prism software (Graph pad, San Deigo, CA). The *K*_*cat*_ was calculated by using the formula *K*_*cat*_ =  *V*_*max*_/[E].

### Homology modeling

The homology model of *Lip* and mutants was searched by SWISS-MODEL (http://swissmodel.expasy.org/) [48]. Based on the model, docking analyses of substrate with *Lip* and mutants were performed by MOE2009 (Chemical Computing Group Inc., Montreal, Canada).

## Additional file

**Additional file 1: Figure S1.** Substrate specificity of purified Y193C. The activity towards *p*-NP acetate (C2) as 100%.

## Abbreviations

*E. coli*: *Escherichia coli*
C2: *p*-nitrophenyl acetate (*p*NPA)
C4: *p*-nitrophenyl butyrate (*p*NPB)
C6: *p*-nitrophenyl hexanoate (*p*NPH)
C8: *p*-nitrophenyl caprylate (*p*NPC)
C12: *p*-nitrophenyl laurate (*p*NPL)
C16: *p*-nitrophenyl palmitate (*p*NPP)
IPTG: isopropyl-β-d-thiogalactopyranoside
PBS: phosphate buffer saline
GST: glutathione S-transferase
