# Supplementary material for: A novel cold-adapted esterase from Enterobacter cloacae: Characterization and improvement of its activity and thermostability via the site of Tyr193Cys
Source: Microb Cell Fact. 2018 Mar 19;17:45. doi: 10.1186/s12934-018-0885-z (PMC5858142; doi:10.1186/s12934-018-0885-z)
Supplement: Supplementary file 1 — Additional file 1: Figure S1. Substrate specificity of purified Y193C. The activity towards p-NP acetate (C2) as 100%. [file 12934_2018_885_MOESM1_ESM.docx]

A novel cold-adapted esterase from *Enterobacter cloacae*: Characterization and improvement of its activity and thermostability via the site of Tyr193Cys

Haofeng Gao^1,2^, Chanjuan Li^3^, Ramesh Bandikari^3^,Ziduo Liu^3^, Nan Hu^2*^, Qiang Yong^1*^

**^1^** College of Light Industry Science and Engineering, Nanjing Forestry University, Nanjing 210018, China

^2^ College of Biotechnology and Pharmaceutical Engineering, Nanjing Tech University, Nanjing 211800, China

^3^College of Life Science and Technology, State key Laboratory of Agricultural Microbiology, Huazhong Agricultural University, Wuhan 430070, China


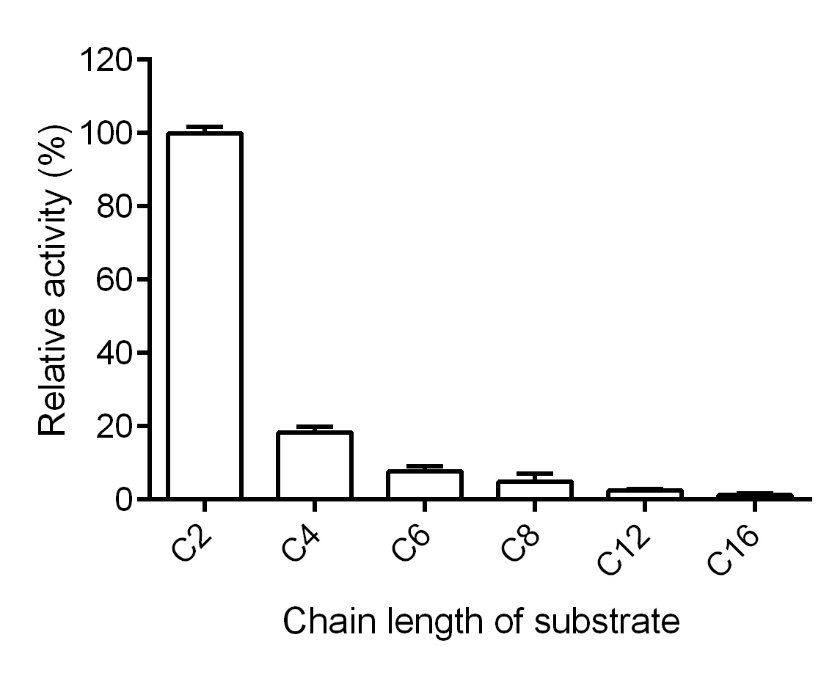


**Figure S1.** Substrate specificity of purified Y193C. The activity towards *p*-NP acetate (C2) as 100%
